# STEM exam performance: Open‐ versus closed‐book methods in the large language model era

**DOI:** 10.1111/tct.13839

**Published:** 2024-11-04

**Authors:** Rasi Mizori, Muhayman Sadiq, Malik Takreem Ahmad, Anthony Siu, Reubeen Rashid Ahmad, Zijing Yang, Helen Oram, James Galloway

**Affiliations:** ^1^ GKT School of Medicine, Faculty of Life Sciences & Medicine King's College London London UK; ^2^ Brighton And Sussex Medical School Sussex UK

## Abstract

**Background:**

The COVID‐19 pandemic accelerated the shift to remote learning, heightening scrutiny of open‐book examinations (OBEs) versus closed‐book examinations (CBEs) within science, technology, engineering, arts and mathematics (STEM) education. This study evaluates the efficacy of OBEs compared to CBEs on student performance and perceptions within STEM subjects, considering the emerging influence of sophisticated large language models (LLMs) such as GPT‐3.

**Methods:**

Adhering to PRISMA guidelines, this systematic review analysed peer‐reviewed articles published from 2013, focusing on the impact of OBEs and CBEs on university STEM students. Standardised mean differences were assessed using a random effects model, with heterogeneity evaluated by *I* ^2^ statistics, Cochrane's *Q* test and Tau statistics.

**Results:**

Analysis of eight studies revealed mixed outcomes. Meta‐analysis showed that OBEs generally resulted in better scores than CBEs, despite significant heterogeneity (*I* ^2^ = 97%). Observational studies displayed more pronounced effects, with noted concerns over technical difficulties and instances of cheating.

**Discussion:**

Results suggest that OBEs assess competencies more aligned with current educational paradigms than CBEs. However, the emergence of LLMs poses new challenges to OBE validity by simplifying the generation of comprehensive answers, impacting academic integrity and examination fairness.

**Conclusions:**

While OBEs are better suited to contemporary educational needs, the influence of LLMs on their effectiveness necessitates further study. Institutions should prudently consider the competencies assessed by OBEs, particularly in light of evolving technological landscapes. Future research should explore the integrity of OBEs in the presence of LLMs to ensure fair and effective student evaluations.

## 1 INTRODUCTION

In the rapidly evolving educational landscape, the methodologies used to assess student competencies are undergoing significant transformations. This is particularly evident within science, technology, engineering, arts and mathematic (STEM) fields, where critical thinking and problem‐solving skills are paramount. Traditionally, educational assessments have been dominated by two primary formats: open‐book examinations (OBEs) and closed‐book examinations (CBEs), each serving distinct pedagogical purposes. CBEs are a traditional form of assessment that has been widely used in educational settings to evaluate students' abilities to recall information from memory.^1^ OBEs, in contrast, permit the use of external resources, thereby assessing students' abilities to synthesise and apply information.^2^ While CBEs are often understood as an assessment method that tests rote memorisation, they can also be structured to evaluate higher‐order cognitive skills. Examples of the various CBEs types include standardised tests (commonly used for uniform evaluation), problem‐solving or case‐based examinations (used to assess the application of knowledge to real‐world scenarios), cumulative exams (assessing entire course material), essay exams (used to assess critical thinking and communication skills) and formula‐based exams (focused on specific formulas or equations).^3^

The unprecedented COVID‐19 pandemic compelled a rapid shift in teaching and assessment methodologies, profoundly impacting educational systems worldwide. *As educational institutions abruptly transitioned to remote learning and OBEs, the crisis led to an extensive re‐evaluation of traditional examination methods*. The transition has spotlighted the efficacy and fairness of OBEs as compared to their closed‐book counterparts, raising questions about their comparability to CBEs in assessing student competency across STEM disciplines. With the presence of viva and objective structured clinical examinations (‘OSCEs’) in disciplines of STEM, which are significantly more difficult to deliver in an OBE format, it has become pertinent to appropriately address the differences in OBEs and CBEs in assessing students in the rapidly changing technological environment. Although OBEs and CBEs may not be able to test interpersonal skills in the same way that OSCEs and viva‐style examinations can, OBEs and CBEs can assess other skills (such as knowledge recall, critical thinking and analysis), and hence, it is important to assess how exam performance varies between these exam structures.

As educational institutions abruptly transitioned to remote learning and OBEs, the crisis led to an extensive re‐evaluation of traditional examination methods.

*The introduction of advanced technologies, particularly Large Language Models (LLMs) like GPT‐3, has further complicated the dynamics of educational assessment methods*. These technologies, capable of generating human‐like text and providing answers to a range of questions, present fresh challenges among educational institutions in their impact on the validity of traditional assessment methods. There is a growing concern that the rapid growth of such technologies could ultimately alter the very competencies being assessed, shifting the focus from information memorisation and application to information retrieval and use.

The introduction of advanced technologies, particularly Large Language Models (LLMs) like GPT‐3, has further complicated the dynamics of educational assessment methods.

For clinical educators, understanding these shifts is crucial. As future health care professionals must navigate a technologically advanced environment, the relevance of assessments that accurately reflect real‐world applications of knowledge becomes paramount. It is imperative that educational institutions and their assessment methods keep up with technological advancements, to ensure that students are adequately and effectively prepared for the challenges of modern clinical practice. In a world of evolving pedagogical advancements, it is also increasingly imperative that ‘traditional’ exam delivery methods are assessed to understand if and how they can be better suited to the changing needs of examinees.

While prior reviews do exist, they are mainly focused on specific health care fields (^4^, ^5^, ^6^) and did not extensively cover STEM disciplines known for their shared foundational tenets. Our systematic review seeks to critically evaluate the effectiveness of OBEs in comparison to CBEs, with a focus on exam performance across a broad range of STEM subjects, with a particular focus on the implications of these findings for clinical education. This review also aims to bridge that gap through an understanding of comparability between OBEs and CBEs in terms of examination achievements as well as students' experiences and perceptions of the respective examination methods.

### 1.1 Research questions

Our study is guided by the following research questions:

1. How OBEs and CBEs differ in terms of student exam performance in STEM subjects?
2. How do students perceive OBEs in comparison to CBEs regarding their learning experience?
3. If discrepancies are observed, what are potential justifications, and what are the implications of these justifications?

By addressing these questions, this review aims to provide valuable insights into the current assessment practices and their alignment with educational goals in a rapidly changing technological environment.

## 2 METHODS

### 2.1 Eligibility criteria

Aligned with PRISMA guidelines, our review focused on peer‐reviewed English articles from 2013 onwards examining OBE versus CBE impacts on university STEM students. This timeframe captures educational shifts pre‐ and during COVID‐19. Detailed course specifics are provided in Appendix S1.

### 2.2 Search strategy and data extraction

We conducted systematic searches in PubMed, Scopus and ERIC from February to May 2023, detailed in the supporting information appendix. Titles and abstracts were independently screened by two reviewers using Rayyan QCRI, with conflicts resolved by a third reviewer. This step categorised studies as ‘exclude’, ‘uncertain’, or ‘include’. Full texts of potentially relevant studies were examined, and their bibliographies were checked for additional eligible studies.

### 2.3 Quality assessment

The quality and risk of bias assessment of the studies included in the analysis were conducted using the Newcastle–Ottawa Scale (NOS) for non‐randomised studies. The NOS is a widely recognised tool that evaluates studies based on three main criteria: selection, comparability, and outcome.^7^ The selection criteria encompass the representativeness of the exposed and non‐exposed cohorts, sample size and ascertainment of exposure. This carries a max score of 5. Comparability focuses on controlling confounding variables, with a max score of 2. Finally, outcome criteria consider the assessment of the outcome and of the statistical test selection, carrying a maximum score of 3. Thus, the studies in this paper were scored on their risk of bias (ROB), from 0 to 10 and also qualitatively, from Low to Medium.

The results of this ROB assessment can be seen in the supporting information Appendix S2.

The assessment process involved two independent reviewers who conducted the evaluations and then resolved any discrepancies through discussion afterwards, thus resulting in a robust and reliable evaluation.

### 2.4 Meta‐analysis methodology

#### 2.4.1 Data synthesis and analysis

We combined data across studies using a random effects model, expecting a high degreee of heterogeneity between studies. To compare results across studies with different scoring systems, standardised mean differences (SMD) in exam scores, scaled from 0 to 100, were calculated. We then computed an overall summary mean difference with 95% confidence intervals (CI) to compare exam performance between OBE and CBE settings.

#### 2.4.2 Assessment of heterogeneity

Heterogeneity, defined as variation in study outcomes that cannot be attributed to chance alone, was assessed with three metrics—the *I* ^2^ statistic, Cochrane's *Q* test and Tau statistic using the restricted maximum likelihood method (REML)^8^.^9^ The presence of heterogeneity (*p*‐value <0.1 from Q test) that is likely contributed by factors other than sampling error (*I* ^2^ > 75%) prompted further exploration via sub‐group analysis by study design (observational versus experimental). Assuming a normal distribution of true effect sizes, the Tau (Τ) value could be interpreted as the standard deviation of the true effect sizes (mean score difference) across studies.

Publication bias was assessed with funnel plot inspections. Significance was set at a *P‐*value of less than 0.05 for all statistical tests. Analyses were conducted using *R* software (version 4.3.1).

## 3 RESULTS

### 3.1 Study selection and characteristics

**FIGURE 1:**
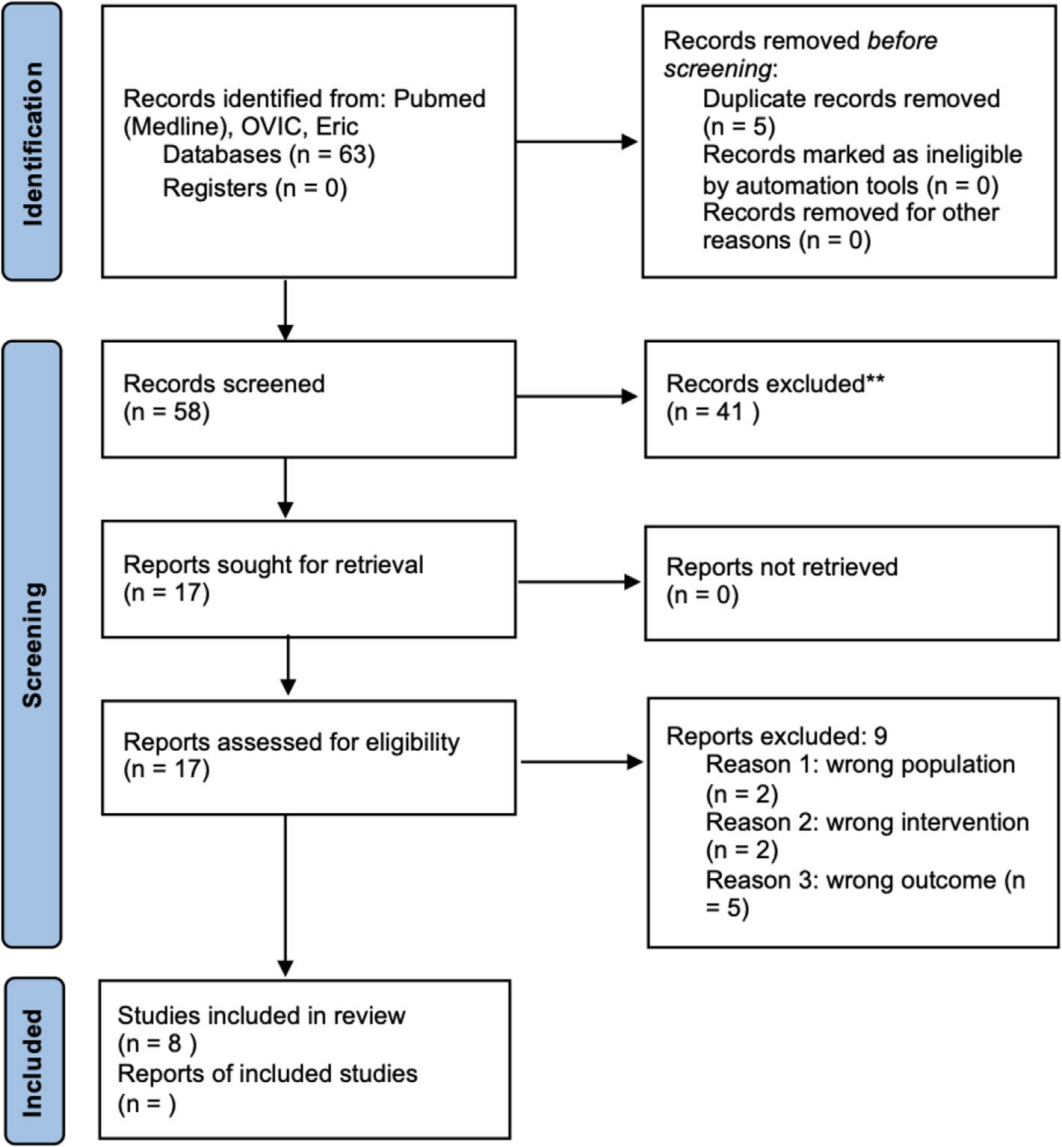
PRISMA flow chart. PRISMA flow diagram demonstrating the process for inclusion of selected papers in the review*.*

The PRISMA flow chart is shown in Figure 1. Out of the eight studies reviewed, all measured exam scores as the primary outcome, with one study also examining time to completion as an additional variable. These studies were single‐institutional, predominantly conducted in the USA (*n =* 5), followed by the UK (*n =* 2) and Saudi Arabia (*n =* 1). They encompassed a range of STEM disciplines, including Medicine, Psychology, Chemistry, Biochemistry and Anatomy, providing a quantitative analysis of exam scores, with some also reporting on completion time and percentage scores.

The design of these studies was heterogeneous; however, they shared the common objective of evaluating student scores in both OBE and CBE settings. Despite this mix, a predominant method was the computer‐delivered format for exams, with variations in proctoring arrangements.

### 3.2 Study quality and bias assessment

The risk of bias was assessed using the Newcastle–Ottawa scale by TM, with MR reviewing the outcomes to resolve any discrepancies through discussion (see Appendix S2). Study quality varied, with only three studies scoring seven or above out of 10. Strengths included robust sample sizes in studies by Erlich et al.^10^ and Davies et al.^11^ enhancing statistical validity and generalisability. Clarity in exposure measurements was another strength, as seen in the contrasting assessment methods in Erlich's study and Jaap et al.'s^12^ description of transitioning to remote exams.

Conversely, weaknesses were noted in the clarity of statistical methods, with papers by Wilhelm et al.^13^ and Balasubramanian et al.^14^ lacking detail. Additionally, control for confounders was insufficient in several studies, notably in Mohanna and Patel's^15^ work, which did not thoroughly address variables such as technical proficiency or pandemic‐related factors. Consistency in exam difficulty and invigilation practices was also unclear, impacting comparability and reliability.

### 3.3 Narrative review

Mixed results were found regarding the impact of OBE on performance compared to CBE. Three studies reported no significant difference in exam scores. However, differences in performance did emerge in some studies under specific conditions, such as the type of questions or exam format changes.

### 3.4 Meta‐analysis findings

#### 3.4.1 Headline findings

**FIGURE 2:**
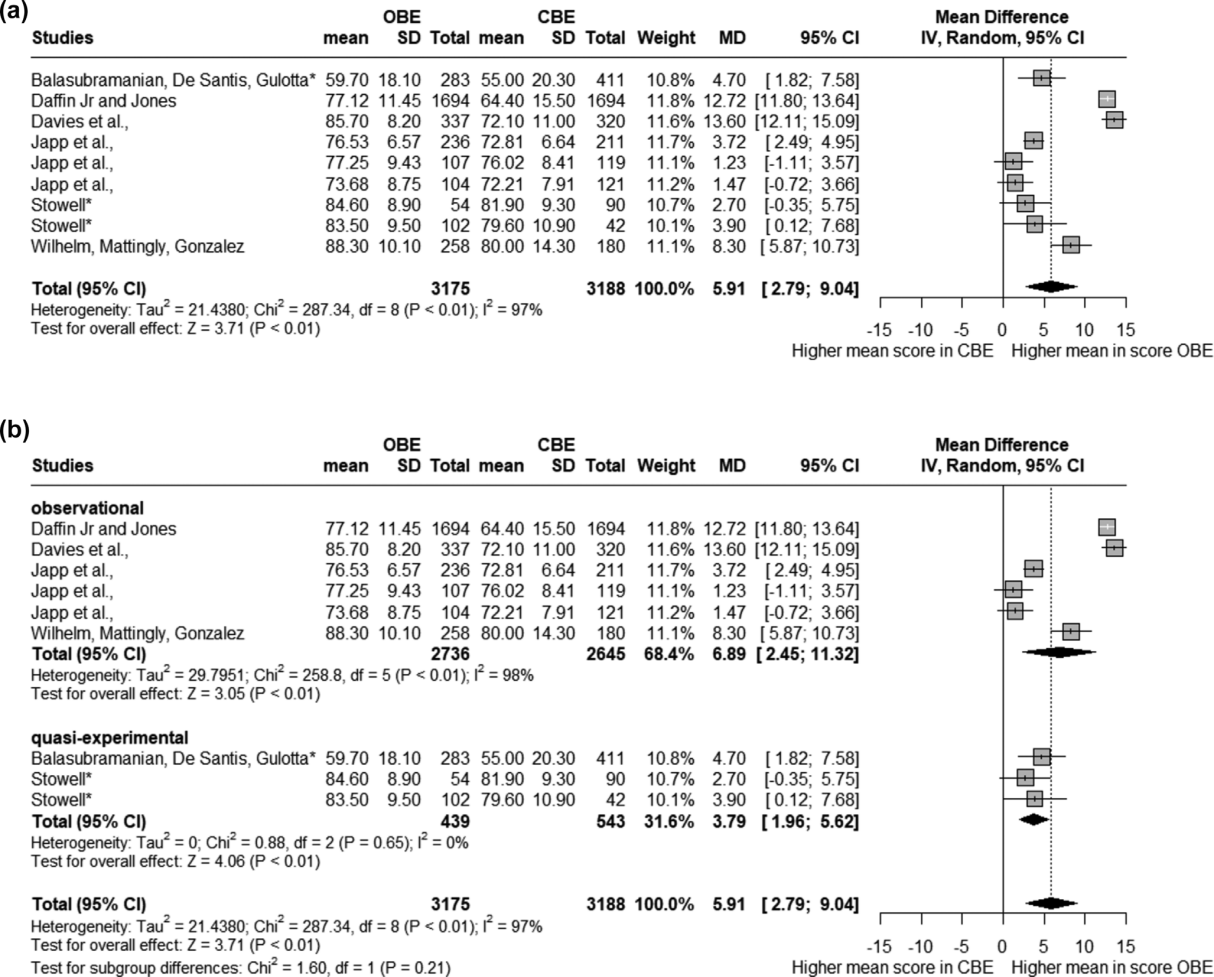
(a) Main meta‐analysis. Overall forest plot of mean exam scores in closed book settings versus open book settings shows a significant difference (*p* < 0.01). CBE, closed book exam; CI, closed book exam; IV, inverse variance weighted; MD, mean difference; OBE, open book exam. (b) Sub‐group meta‐analysis plot. Forest plot divided into sub‐groups based on study design. No significant sub‐group difference is noted by the Chi^2^ test (*p* = 0.21*).*

*The meta‐analysis revealed a notable increase in marks for OBEs compared to CBEs (see* Figure 2a *), with an overall mean difference of 5.91 (95% CI: 2.79 to 9.04*). This suggests that students scored higher in open‐book exam settings relative to closed‐book settings.

#### 3.4.2 Analysis of heterogeneity

*There was substantial heterogeneity in the meta‐analysis results, indicated by an I* ^*2*^ *value of 97%*. This reflects significant variability in the study outcomes.

#### 3.4.3 Sub‐group analyses

Further investigation via sub‐group analysis (see Figure 2b) showed that the mean difference for observational studies was 6.89 (95% CI: 2.45 to 11.32; *I* ^2^ = 98%), indicating a strong effect favouring OBEs. Quasi‐experimental studies presented a mean difference of 3.79 (95% CI: 1.96 to 5.62; *I* ^2^ = 0%), suggesting a more modest but still significant benefit of OBEs over CBEs. Notably, a Chi‐square test for sub‐group differences showed no statistically significant variance in effect sizes between observational and quasi‐experimental studies (Chi^2^ *p* = 0.21).

Jr et al.'s^16^ study highlighted the negative impact of proctoring on performance and time taken to complete exams. In contrast, Balasubramanian et al. did not find significant differences in exam performance due to proctoring. As well as the effects of proctoring, technical challenges were also common, affecting performance and completion of online examinations. Problems ranged from hardware limitations to internet connectivity, sometimes leading to early submission and affecting student performance. Cheating was also reported in the study by Mohanna and Patel's study, with distinct patterns observed in open‐book and invigilated exams. Measures to reduce cheating were noted, including variability in exam questions.

## 4 DISCUSSION

The current evidence base that compares OBE and CBE is both narrow and heterogeneous, making generalisability challenging. Despite this, our meta‐analysis, which draws from a diverse, albeit small, pool of studies, indicates that *OBEs are associated with 6% higher marks than CBEs*. This finding persisted across observational and quasi‐experimental designs. However, the underlying reasons for this difference remain elusive, as our analysis could not conclusively attribute it to a specific factor such as cheating, proctoring practices or technical delivery methods. Consequently, we infer that OBEs and CBEs likely assess distinct competencies.

OBEs are associated with 6% higher marks than CBEs.

*The integration of LLMs like GPT‐3 into the examination landscape brings both opportunities and significant challenges that necessitate a re‐evaluation of current testing paradigms in both OBE and CBEs*. Although OBEs may once have been considered advantageous due to the reduced need for invigilation, the advent of AI technologies such as OpenAI presents fresh challenges to credibility and integrity of test results, especially when these assessment are delivered online. Given that AI models can readily answer short‐answer questions that test memory recall, potentially discouraging students from developing their information‐searching skills, *LLMs have the potential to inflate scores by enabling students to answer questions without a deep understanding of the subject*.^17^ This is particularly problematic in unproctored environments, where the use of AI tools can go unchecked, leading to questions about the fairness and validity of the assessments.^18^ Consequently*, the shift towards proctored examinations or the development of countermeasures to mitigate these risks is becoming increasingly necessary*.

LLMs have the potential to inflate scores by enabling students to answer questions without a deep understanding of the subject.

In response to these challenges, it is now understood that the test design has a significant influence on the impact of LLMs. The poor performance of LLMs on certain intelligence tests, for example biomedical natural language processing tasks^19^ is a testament of the impact that test design can have. *Test questions may need to evolve from traditional formats to more complex, application‐based scenarios that LLMs struggle to navigate effectively*. This might include the integration of questions that require a higher order of cognitive processing or practical application, which LLMs are currently less equipped to handle.^20^ While this is the current situation, with the rapid development of AI models, the assistance provided by LLMs will only become more sophisticated with time and has the potential to eventually undermine the skills intended to be assessed by OBEs, extending beyond simple recall to clinical reasoning. While OpenAI and ChatGPT may not be able to support examinees to the same extent for open‐ended and essay‐style questions, there is potential.^21^ It is, therefore, crucial that educational institutions and faculties bolster their capabilities to support these advanced testing formats, ensuring secure testing platforms and equitable access to necessary technologies for all students.^22^ As well as bolstering its infrastructure, institutions must also focus on quality assurance processes to maintain the validity and fairness of assessments Otherwise, without the correct action, as these LLM tools become more prevalent, the risk is that they may reduce the need for student to engage deeply with the material, thereby diminishing the value of OBEs in assessing genuine understanding and compromising the distinct value and benefits of OBEs compared to CBEs, rendering the differentiation between the 2 examination methods insignificant.^21^

Test questions may need to evolve from traditional formats to more complex, application‐based scenarios that LLMs struggle to navigate effectively.

To add to this, in considering the integration of LLMs within curriculums such as medical ones, it is crucial to acknowledge their inherent limitations alongside their benefits. LMM's such as GPT‐3, even in the absence of specialist training or reinforcement, store a breadth of medical knowledge which can prove threatening to the integrity of medical school examinations in an open book setting. For example, in 2023, Kung and colleagues investigated the use of ChatGPT to write the USMLE Step 1, Step 2CK and Step 3 examinations which are used to assess medical students in the United States, and without any specialised reinforcement was able at or near to the passing threshold of each of the three assessments independently.^23^ As such models continue to learn and improve, their use in medical education must be considered but controlled, and in situations where a medical student's knowledge base needs to be examined, there should be restrictions on the use of LLMs to protect the integrity of the assessment.

On the other hand, while proficient in processing and generating vast amounts of information, LLMs play a considerably less significant role in developing essential interpersonal skills that are critical for medical practitioners. These skills include interpersonal interactions, procedural capabilities as well as the integration of theoretical knowledge into practical, simulated scenarios typical in Objective Structured Clinical Examinations (OSCEs). As such, the reliance on LLMs for this aspect of medical training is minimal. This delineation is essential within curriculum planning, because while students can leverage the analytical and informational capabilities of LLMs within certain assessment methods, other assessment methods, like OSCEs, necessitate concurrent development of the hands‐on and interpersonal skills that are crucial for their success in the exams and their future clinical practice.

*OBEs present considerable logistical advantages for academic institutions by reducing the resource and financial burdens associated with traditional invigilated exams*. Furthermore, student preferences lean towards OBEs, citing reduced stress^24^ and a desire for less intrusive exam oversight, potentially fostering a more positive attitude towards assessments. It may also be argued that OBEs are fairer as they are less reliant on excellent memorisation abilities, and instead require a more comprehensive understanding and the ability to apply knowledge. Notable, though, is that similar studies have demonstrated the need for faculty training and adequate technological support.^25^ These factors, combined with the need for assessments to mirror real‐world information access, push the conversation towards redefining the objectives and delivery of exams in academic settings.

OBEs present considerable logistical advantages for academic institutions by reducing the resource and financial burdens associated with traditional invigilated exams.

The preference for OBEs among students and the emphasis on assessments reflecting real‐world practices cannot be ignored. *In an age where instantaneous access to information is the norm, the pedagogical value of memorising information is increasingly questioned*. However, there is an evidence gap regarding whether a deeper reservoir of memorised knowledge correlates with better professional performance compared to the ability to search for information online efficiently. At present, data on this question are absent from the evidence base.

In an age where instantaneous access to information is the norm, the pedagogical value of memorising information is increasingly questioned.

### 4.1 Strengths and limitations

Our review stands as the most comprehensive synthesis of existing data comparing OBE and CBEs within STEM disciplines. By encompassing a range of subjects within this field, it enhances the robustness of the findings and provides a broad empirical basis for conclusions. This expansive approach across various STEM subjects allows for a more nuanced understanding of the impacts of examination type on student performance, offering valuable insights for educators and policymakers.

Despite these strengths, there are significant limitations to our study. The evidence base is relatively small, with included studies originating from only three countries, potentially neglecting the representation of the global academic environment. There is a substantial degree of uncertainty in our findings; the lower bound of our primary analysis confidence interval extends to a difference that could be considered marginally important, indicating that the true effect size might be smaller than the mean difference suggests.

*A further critical limitation is that all the studies we reviewed were conducted before the widespread introduction of major LLMs*, such as GPT‐3. This omission is significant because the capabilities of LLMs, particularly in terms of information synthesis and problem‐solving, could dramatically alter the landscape of OBEs. The absence of data from the LLM era means our conclusions might not fully encapsulate the current challenges and advantages associated with OBEs. Looking ahead, as medical schools increasingly consider a long‐term shift towards OBEs, it becomes essential to deliberate on how students are permitted to access these resources. For example, while students may be allowed to access the internet to retrieve useful information, restrictions might be necessary to prevent direct access to LLMs that could autonomously provide answers, thus maintaining the integrity and intended learning outcomes of the assessments.

## 5 FUTURE RECOMMENDATIONS.

*As LLMs continue to evolve and influence educational methodologies, the assumptions underlying both OBEs and CBEs may need to be re‐evaluated* to ensure they are still achieving their intended pedagogical objectives. Although LLMs are still in their nascent stages, initial stages, the implications are profound, warranting further research into their impact on OBEs. Future research should investigate the impact that the expansion of LLMs can have on OBEs, ensuring that students' use of LLMs is constructive and does not compromise the benefits that OBEs offer compared to CBEs.

In addition, should there be a widespread consideration of a shift towards OBEs in the future, research must be undertaken into the integrity of the exams with respect to AI usage. With models such as ChatGPT, short‐answer questions being asked in OBEs may be easily answered, whereas where longer‐answer questions and essays require more critical thinking requiring more critical thinking, ChatGPT may not be equally useful. This poses the question of whether open‐book style assessments should be utilised for long‐answer questions which focus on deep‐thinking which can be supported with information online as opposed to memory recall, whereby facts can be quickly found using LLMs. For short‐answer and ‘recall’ style questions, either a closed‐book model or an open‐book with restrictions on the use of AI models (hence requiring invigilation) should be used. OBEs would still serve useful as a way of improving students' efficiency at finding information online but would reduce reliance on AI to do the information‐searching for them completely.

Provided this information, course coordinators and alike for medical school programmes should consider the ethics behind OBEs given the rise of AI; understanding the implications of the use of AI in OBEs within medical education, and how students decide to use AI when undertaking assessments in this style. Moreover, future research should look into whether a combined approach to assessments an appropriate way of advancing would be. For instance, consider an essay‐style question where students could initially be allowed access to AI and the internet for a defined period to gather the necessary factual information. Subsequently, the examination could shift to a closed‐book format, requiring students to independently synthesis, culminate the breadth of information and articulate their understanding in their responses.

## 6 CONCLUSION

The higher marks associated with OBEs suggest that this assessment format is not simply an alternative to CBEs but a different metric of student ability, potentially emphasising skills more attuned to the current and future educational landscapes. However, the limited generalisability of our results necessitates caution. Institutions considering the shift towards OBEs must do so with a clear understanding of the competencies they aim to measure and develop. As this shift is considered, they must consider the authentic aspect of OBEs compared to CBEs and how this authentic aspect may be rendered insignificant by the rapid growth of LLMs. As educational paradigms shift, a balanced approach may be warranted, one that integrates the strengths of both OBEs and CBEs to provide a holistic assessment of student competencies. With a constantly evolving technological climate and increasing use of AI models such as ChatGPT, it raises the question on if OBEs should be used for specific types of examinations depending on the style of question being used.

## AUTHOR CONTRIBUTIONS

**Rasi Mizori:** Writing—original draft; writing—review and editing; data curation; conceptualization. **Muhayman Sadiq:** Writing—original draft. **Malik Takreem Ahmad:** Writing—original draft; data curation; methodology. **Anthony Siu:** Writing—original draft; formal analysis; data curation. **Reubeen Rashid Ahmad:** Formal analysis. **Zijing Yang:** Formal analysis. **Helen Oram:** Writing—review and editing; supervision. **James Galloway:** Writing—review and editing; conceptualization; supervision; validation.

## CONFLICT OF INTEREST STATEMENT

The authors declare no potential conflicts of interest with respect to the publication, research, and/or authorship of this article.

## ETHICS STATEMENT

This research study adheres to the highest standards of academic integrity and ethical research practices. The review does not engage with human participants or collect primary data. All sources have been duly cited and acknowledged in line with academic guidelines to maintain transparency and integrity in the research process. Consequently, there are no ethical concerns associated with this work.

## Supporting information


**APPENDIX S1:** Data extraction table for the 8 studies included in the paper.
APPENDIX S2: Risk of bias table demonstrating results obtained using the Newcastle‐Ottawa Scale to assess papers involved in the study.
APPENDIX S3: Funnel plot demonstrating publication bias found within the involved studies.

## Data Availability

As a systematic review, the study analyses eight scholarly papers sourced from digital databases and online journals. Some articles were accessed via public domain resources, while others required institutional access through the university I study at: King's College London. Accessibility of these papers varies according to each journal's policy and the reader's access privileges. For details on each paper's accessibility and full citations, please see the References section of this manuscript.

## References

[1] Theophilides C , Koutselini M . Study behavior in the closed‐book and the open‐book examination: a comparative analysis. Educ Res Eval. 2000;21(1):379–393. Available at: 10.1076/EDRE.6.4.379.6932

[2] Krasne S , Wimmers PF , Relan A , Drake TA . Differential effects of two types of formative assessment in predicting performance of first‐year medical students. Adv Health Sci Educ. 2006;11(2):155–171. 10.1007/S10459-005-5290-9/METRICS 16729243

[3] Agarwal PK , Karpicke JD , Kang SHK , Roediger HL III , McDermott KB . Examining the testing effect with open‐ and closed‐book tests. Appl Cogn Psychol. 2008;22(7):861–876. 10.1002/ACP.1391

[4] Dave M , Patel K , Patel N . A systematic review to compare open and closed book examinations in medicine and dentistry. Fac Dent J. 2021;12(4):174–180. 10.1308/rcsfdj.2021.41

[5] Durning SJ , Dong T , Ratcliffe T , Schuwirth L , Artino AR Jr , Boulet JR , et al. Comparing open‐book and closed‐book examinations: a systematic review. Acad Med. 2016;91(4):583–599. 10.1097/ACM.0000000000000977 26535862

[6] Johanns B , Dinkens A , Moore J . A systematic review comparing open‐book and closed‐book examinations: evaluating effects on development of critical thinking skills. Nurse Educ Pract. 2017;27:89–94. 10.1016/J.NEPR.2017.08.018 28881323

[7] Stang A . Critical evaluation of the Newcastle‐Ottawa scale for the assessment of the quality of nonrandomized studies in meta‐analyses. Eur J Epidemiol. 2010;25(9):603–605. 10.1007/S10654-010-9491-Z/TABLES/1 20652370

[8] Higgins JP , Thompson SG , Deeks JJ , Altman DG . Measuring inconsistency in meta‐analyses. BMJ: Brit Medl J. 2003;327(7414):557. 10.1136/BMJ.327.7414.557 PMC192859 12958120

[9] Tanriver‐Ayder E , Faes C , Van de Casteele T , McCann SK , Macleod MR . Comparison of commonly used methods in random effects meta‐analysis: application to preclinical data in drug discovery research. BMJ Open Sci. 2021;5(1). 10.1136/BMJOS-2020-100074 PMC8647574 35047696

[10] Erlich, D. Because life is open book: an open internet family medicine clerkship exam, PRiMER (Leawood, Kan.), 1. 2017 Available at: 10.22454/PRIMER.2017.626578 PMC7490191 32944693

[11] Davies DJ , McLean PF , Kemp PR , Liddle AD , Morrell MJ , Halse O , et al. Assessment of factual recall and higher‐order cognitive domains in an open‐book medical school examination. Adv Health Sci Educ. 2022;27(1):147–165. 10.1007/S10459-021-10076-5 PMC8536902 34687383

[12] Jaap A , Dewar A , Duncan C , Fairhurst K , Hope D , Kluth D . Effect of remote online exam delivery on student experience and performance in applied knowledge tests. BMC Med Educ. 2021;21(1):86. 10.1186/s12909-021-02521-1 33530962 PMC7851803

[13] Wilhelm J , Mattingly S , Gonzalez VH . Perceptions, satisfactions, and performance of undergraduate students during Covid‐19 emergency remote teaching. Anat Sci Educ. 2022;15(1):42–56. Available at: 10.1002/ASE.2161 34859608 PMC9011711

[14] Balasubramanian B , Desantis C , Gulotta M . Assessment à la mode: implementing an adaptable large‐scale multivariant online deferred‐grade exam for virtual learning. J Chem Educ. 2020;97(12):4297–4302. 10.1021/ACS.JCHEMED.0C00767

[15] Mohanna, K. and Patel, A. ‘Overview of open book‐open web exam over blackboard under e‐learning system’, Proceedings ‐ 2015 5th International Conference on e‐Learning, ECONF, 2015, pp. 396–402. Available at: 10.1109/ECONF.2015.81

[16] Daffin LW , Jones AA . Comparing student performance on proctored and non‐proctored exams in online psychology courses. Online Learning. 2018;22(1):131–145. 10.24059/OLJ.V22I1.1079

[17] Park YJ , Pillai A , Deng J , Guo E , Gupta M , Paget M , Naugler C. Assessing the research landscape and clinical utility of large language models: a scoping review, 2023, Available at: 10.21203/RS.3.RS-3472000/V1 PMC10936025 38475802

[18] Fagbohun O , Iduwe NP , Abdullahi M , Ifaturoti A , Nwanna OM . Beyond traditional assessment: exploring the impact of large language models on grading practices A B S T R A C T journal of artificial intelligence, machine learning and data science. J Artif Intell Mach Learn & Data Sci. 2023;2(1):1–8. 10.51219/JAIMLD/oluwole-fagbohun/19

[19] Feng H , Rough K , Milligan PB , Tombini F , Kwon T , Zine El Abidine K , Mack CD , Hughes B , How well it works: benchmarking performance of GPT models on medical natural language processing tasks, 2024, medRxiv, p. 2024.06.10.24308699. Available at: 10.1101/2024.06.10.24308699

[20] Thistleton, E. and Rand, J. Investigating deceptive fairness attacks on large language models via prompt engineering. 2024, Available at: 10.21203/RS.3.RS-4655567/V1

[21] Dave M , Patel N . Artificial intelligence in healthcare and education. Br Dent J. 2023;234(10):761–764. 10.1038/S41415-023-5845-2 37237212 PMC10219811

[22] Edralin DM , Pastrana RM . Developing an instrument to assess organizational readiness for a sustainable E‐learning in the new Normal. Bedan Res J. 2021;6(1):1–30. 10.58870/BERJ.V6I1.20

[23] Kung TH , Cheatham M , Medenilla A , Sillos C , De Leon L , Elepaño C , et al. Performance of ChatGPT on USMLE: potential for AI‐assisted medical education using large language models. PLOS Dig Health. 2023;2(2):e0000198. 10.1371/JOURNAL.PDIG.0000198 PMC9931230 36812645

[24] Stowell JR . Online open‐book testing in face‐to‐face classes. Scholarsh Teach Learn Psychol. 2015;1(1):7–13. Available at: 10.1037/STL0000014

[25] Er HM , Wong PS , Nadarajah VD . Remote online open book examinations: through the lenses of faculty and students in health professions programmes. BMC Med Educ. 2023;23(1):397. 10.1186/S12909-023-04368-0 37268906 PMC10235823

