# Supplementary material for: STEM exam performance: Open‐ versus closed‐book methods in the large language model era
Source: Clin Teach. 2024 Nov 4;22(1):e13839. doi: 10.1111/tct.13839 (PMC11663729; doi:10.1111/tct.13839)
Supplement: Supplementary file 1 — APPENDIX S1: Data extraction table for the 8 studies included in the paper. APPENDIX S2: Risk of bias table demonstrating results obtained using the Newcastle‐Ottawa Scale to assess papers involved in the study. APPENDIX S3: Funnel plot demonstrating publication bias found within the involved studies. [file TCT-22-e13839-s001.docx]

# Appendices

**APPENDIX 1 – Data extraction table**

*Appendix 1: Data extraction table for the 8 studies included in the paper.*

**APPENDIX 2 – Risk of bias table**

*Appendix 2: Risk of bias table demonstrating results obtained using the Newcastle-Ottawa Scale to assess papers involved in the study.*

**APPENDIX 3 – Publication bias funnel plot**

**
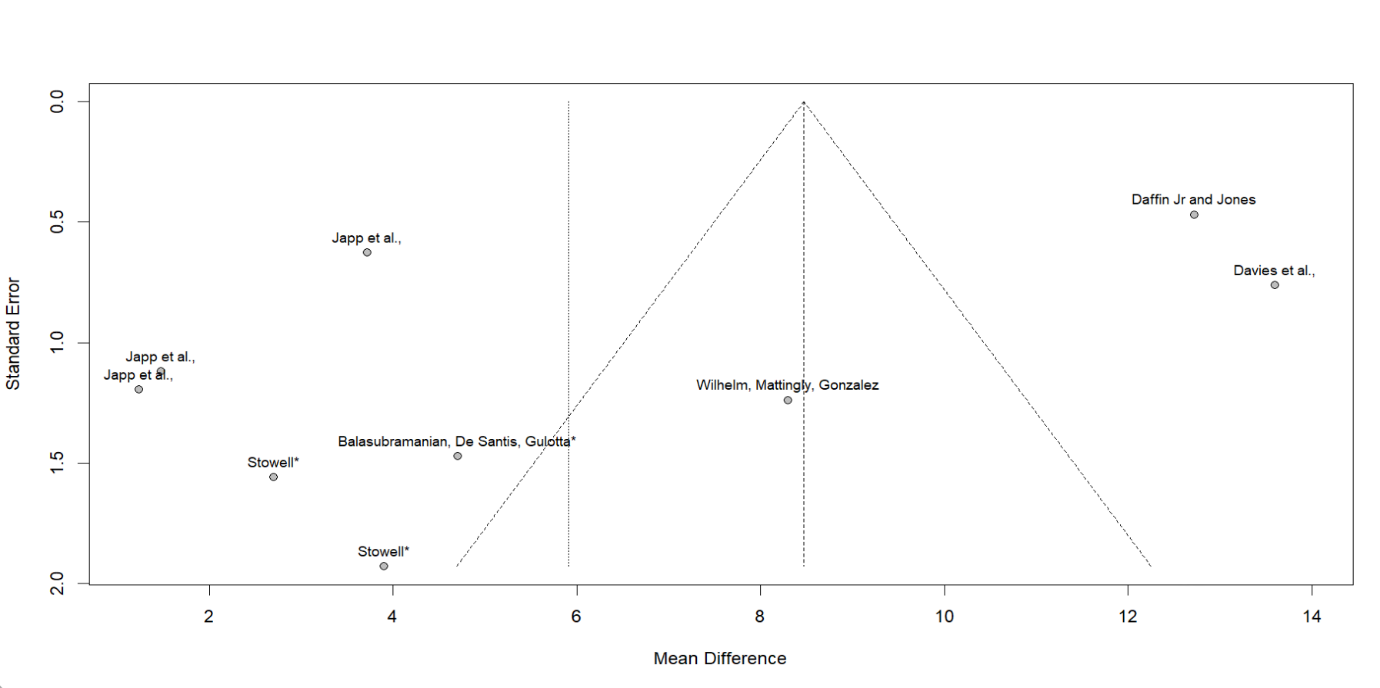
**

*Appendix 3: Funnel plot demonstrating publication bias found within the involved studies.*
